# Paracrine signaling in mammary gland development: what can we learn about intratumoral heterogeneity?

**DOI:** 10.1186/bcr3610

**Published:** 2014-01-29

**Authors:** Jeffrey M Rosen, Kevin Roarty

**Affiliations:** 1 Department of Molecular and Cellular Biology, Baylor College of Medicine, One Baylor Plaza, Houston TX 77030, USA

## Abstract

Paracrine signaling mechanisms play a critical role in both normal mammary gland development and breast cancer. Dissection of these mechanisms using genetically engineered mouse models has provided significant insight into our understanding of the mechanisms that guide intratumoral heterogeneity. In the following perspective, we briefly review some of the emerging concepts in this field and emphasize why elucidation of these pathways will be important for future progress in devising new and improved combinatorial therapeutic approaches for breast and other solid cancers.

## Introduction

One of the basic tenets of developmental biology is that there are signaling cells responsible for generating local factors and target cells that respond to these neighboring cues to regulate cell fate and developmental outcome [1]. These signaling interactions can be between tissue compartments (for example, epithelial-stromal interactions) or within a given tissue compartment (for example, interactions between epithelial cells). Accordingly, cell-cell interactions and paracrine signaling play critical roles in the regulation of tissue morphogenesis, including in mammary gland development [2]. This review is focused on interactions between epithelial cells, although interactions with cells in the microenvironment, especially cells of the immune system, are equally important components of these paracrine regulatory networks (for an excellent review, see [3]).

In systems biology, there has generally been a tendency to overlook these types of interactions and instead to model signal transduction pathways in a cell-autonomous manner. Although there has been increasing emphasis on the interaction of breast cancer cells with their microenvironment, less attention has been placed on understanding the potential of cell-cell and paracrine interactions within the heterogeneous tumor environment. These signaling interactions likely shape the diverse cellular phenotypes that constitute the heterogeneous landscape within individual tumors.

In the past few years, deep sequencing of breast cancers has revealed enormous intratumoral heterogeneity [4]. This heterogeneity has been hypothesized to arise in part through branched Darwinian evolution of genetically diverse subclones that arise during tumor progression [5]. Single-cell analysis has revealed even greater genetic diversity and supports the idea that tumors progress by punctuated clonal expansions [6]. These diverse clones display variability with respect to both their tumor propagation ability and responses to therapy [7]. Tumors that display the greatest degree of genetic instability are also often the most refractory to treatment [8]. In addition to genetic diversity, the varied functional properties of cellular subpopulations within tumors are influenced by epigenetic factors. Though not mutually exclusive with the clonal selection hypothesis, the cancer stem cell hypothesis posits the existence of a self-renewing population of cells that have ‘the developmental potential to recapitulate all the cell types found in a given tissue’ [9]. Furthermore, a ‘niche’ microenvironment is thought to be important in the regulation of stem cell quiescence and differentiation. This niche may be composed of either additional tumor cells or cells from the microenvironment, such as fibroblasts and endothelial cells, or both.

Given that different cellular subpopulations can show large differences in regenerative behavior and treatment response, both within a given tumor and across a collection of tumors, a better understanding of the relationships among the different cell subpopulations within breast cancers will be critical to the development of new and improved therapeutics. Clearly, deciphering the mechanisms involved in this complex biology in breast cancer is a daunting task, especially if (as suggested by deep sequencing) each cancer may be unique. If each tumor follows its own set of growth regulatory rules based on its own cellular makeup and genetic/epigenetic diversity, how can these differences be understood within a unifying context?

An alternative complementary approach to unraveling intratumoral heterogeneity is to better elucidate the behaviors of different cell populations during normal mammary gland development. Unlike breast cancer, in which the cells may have heterogeneous properties due to various mutations, the ‘normal’ luminal and basal mammary epithelial cell types that are composed of stem and progenitor cells should have more predictable developmental behaviors [10]. Thus, as a first step in this process, it would seem self-evident that the best way to approach this problem is to try to understand the interactions between the ligands and receptors in these different compartments during mammary gland development. Finally, since the output of a given signaling pathway (for example, the Wnt and Notch pathways) will *a priori* be cell context-dependent, studies to determine gene expression changes ideally should be performed on defined cell subpopulations. In the following, we will briefly highlight a few of the important concepts and pathways.

## Paracrine signaling pathways and mammary gland development

**Figure 1:**
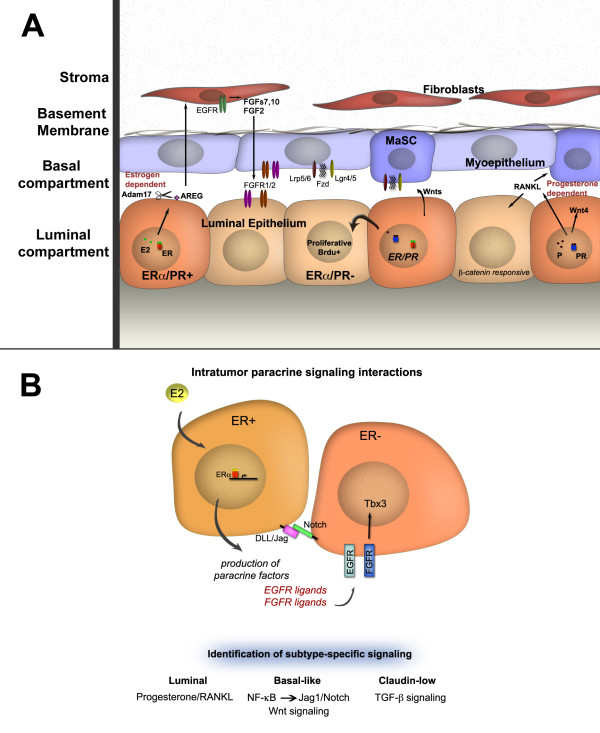
**Signaling interactions in mammary development and cancer. (A)** An abbreviated view of paracrine interactions in mammary development. Estrogen receptor alpha/progesterone receptor-positive (ERα/PR^+^) cells provide extrinsic cues to ERα/PR^-^ cells to enable proliferation. Hormone-specific paracrine mediators involve amphiregulin, which acts downstream of estrogen, and RANKL (receptor activator of nuclear factor kappa-B ligand) and Wnt4, which act downstream of progesterone signaling. Other known paracrine mediators involve Wnts, fibroblast growth factors (FGFs), insulin-like growth factor, bone morphogenetic proteins, transforming growth factor-beta (TGF-β), and Notch; however, owing to space limitations, not all are depicted in this simple model. **(B)** Breast cancer subtypes reveal both inter- and intratumoral heterogeneity. Studies have identified interactions between ERα^+^ cancer cells and ERα^-^ cancer cells involving epidermal growth factor, FGF, and Notch interactions. The identification of signaling crosstalk between cancer cell subpopulations within breast cancer subtypes remains a challenge, yet certain pathways have been identified, including PR/RANKL, Wnt, Notch, and TGF-β. Further studies are required to elucidate the details of subtype-specific differences in paracrine signaling pathways. Cells of the microenvironment (not depicted in this graphic) provide another layer of complexity and play an instrumental role in tumor progression and heterogeneity. EGFR, epidermal growth factor receptor; FGFR, fibroblast growth factor receptor; MaSC, mammary stem cell; NF-κB, nuclear factor kappa-light-chain-enhancer of activated B cell.

The majority of mammary gland development occurs post-natally and is regulated by both systemic hormones and local growth factors. A role for paracrine signaling pathways in mammary gland development was first suggested by studies in which there was a dissociation of the localization of estrogen receptor (ER)-positive and progesterone receptor (PR)-positive cells from proliferative cells in the ductal epithelium of several mammalian species [11-13] (Figure 1A). These descriptive studies were complemented by elegant genetic studies in which mammary gland chimeras were generated by transplanting mixtures of wild-type mammary epithelial cells (MECs) and ER- or PR-null MECs into the cleared fat pads of recipient mice. In both cases, the presence of wild-type cells rescued the null phenotype (that is, the lack of alveologenesis observed with PR-null transplants or the failure to obtain ductal outgrowths with ER-null transplants) [14-16]. These studies represented formal genetic proof that paracrine factors made by the wild-type cells were acting on steroid receptor-null cells to facilitate normal ductal and alveolar morphogenesis. Thus, studies of chimeric outgrowths in the mammary gland are similar but not identical to the kinds of clonal analyses performed in the Drosophila eye to distinguish cell-autonomous from non-autonomous pathways.

A number of paracrine mediators have been identified that might account for the effects of steroid hormones. Receptor activator of nuclear factor kappa-B ligand (RANKL), Wnt 4, and IGF-II were all identified as potential paracrine mediators of PR [17-19], whereas amphiregulin was suggested to be a critical paracrine mediator of ER [20] (Figure 1A). Furthermore, several of these mediators have been shown to be required for progesterone- and estrogen-induced proliferation in the mammary gland both in genetically engineered mouse models [21] and more recently in tissue microstructures isolated from primary human reduction mammoplasties [22]. Thus, these hormonal mechanisms are apparently conserved across species [22]. Mammary stem cells (MaSCs) have also been shown to be sensitive to steroid hormones despite the lack of steroid receptors in MaSCs [23]. Consistent with this observation, progesterone-regulated RANKL and Wnt 4 have been demonstrated to induce mammary stem cell expansion [24]. More recently, progesterone-RANKL paracrine signaling also has been shown to regulate Elf5 expression in luminal progenitors [25]. In these studies, progesterone-induced side branching and the expansion of Elf5^+^ mature luminal cells were prevented by inhibition of RANKL action. Thus, steroid hormone-regulated paracrine mechanisms may affect luminal and basal cells as well as stem and progenitor cells, potentially in both compartments.

Two independent studies performed in our laboratory also illustrate the importance of paracrine signaling mechanisms in the mammary gland. While investigating the role of Hedgehog signaling in the mammary gland, we showed that ectopic expression of the Hedgehog effector protein Smoothened (Smo) is commonly observed in a subset of cells in early breast disease but that these Smo-expressing cells are largely quiescent despite elevated proliferation in the surrounding lesion [26]. When a conditional allele of constitutively active Smo activated by either a mouse mammary tumor virus or adenoviral-Cre recombinase was used, high levels of proliferation were observed in cells adjacent to or in close proximity to but not in the Smo-expressing cells, demonstrating a paracrine induction of proliferation by ectopic SMO expression [27].

In studies of fibroblast growth factor receptor (FGFR) signaling analogous to those performed with the steroid-receptor chimeric outgrowths, deletion of both FGFRs inhibited mammary ductal outgrowths and led to a loss of the basal/MaSC population [28]. Surprisingly, a 10-fold excess of wild-type cells was able to rescue the FGFR1/2-null cells. Intriguingly, in gain-of-function studies, activation of FGFR1 has been shown to rapidly induce amphiregulin expression, and FGFR-induced tumorigenesis was shown to be dependent on epidermal growth factor receptor signaling [29]. Thus, both ER and FGFR appear to regulate amphiregulin expression perhaps in luminal cells. Members of the erbB family of receptors, such as erbB3, have also been shown to be important regulators of the balance of luminal and basal mammary epithelium [30]. ErbB3 is expressed primarily in the luminal epithelium, and deletion in the luminal but not the basal epithelium resulted in growth of the basal cells, in part due to induction of cytokines such as IL-6 in the luminal epithelial cells [30].

One of the most complicated paradigms to understand with respect to mammary gland development and breast cancer is illustrated by the Wnt pathway. There are 19 mammalian Wnt ligands, 10 frizzled receptors, and other non-frizzled tyrosine kinase receptors like Ror1, Ror2, and Ryk, together with co-receptors such as LRP5 and LRP6 that can signal through a variety of downstream pathways. The ligand-receptor context is viewed as a key determinant of whether the Wnt signaling output is through the canonical β-catenin-dependent pathway or alternative non-canonical β-catenin-independent pathways [31,32]. In addition, R-spondins and various leucine-rich repeat containing G protein receptors are expressed and play important roles in mammary gland development, specifically with regard to stem cell function [32-34]. Many studies thus far have centered around Wnt and stem cell function; however, Wnt ligands and receptors exhibit striking specificity in their expression throughout development. These observations highlight the potential differential functions of Wnt pathway components in luminal and basal cells in the mammary gland and even within different subpopulations of cells in breast cancer, specifically where Wnt signatures seem to be evident in basal-like breast cancers [35,36]. Thus, understanding the precise integration of signaling downstream of these ligands and receptors using genetic approaches remains a complicated task.

Wnt signaling is critical for mammary stem cell self-renewal with unique developmental stage and time dependencies [32,37,38]. Intriguingly, recent studies using embryonic stem cells have suggested that localized Wnt signals can orient asymmetric stem cell division [39]. In the mammary gland, localized Wnt signaling most likely plays a critical role in regulating the orientation of cell division and the cellular fate during development. Additionally, as with many signaling pathways, the activity of the signaling event is not indicated simply by an ‘on or off’ state but is instead measured by the level and duration of the signaling [40]. Recent studies suggest that elevated paracrine Wnt signals in the context of tumorigenesis can exert distinct outcomes on the receiving cells within breast xenograft models and emphasize the challenge of deciphering Wnt interactions *in vivo*[41]. An understanding of the regulation of Wnt ligand expression by systemic hormones, the spatial and temporal heterogeneity of ligand and receptor expression, and the cell context dependence of Wnt signaling, therefore, will be required before we will fully elucidate its role in mammary gland development. Modeling these complex cell-cell and paracrine interactions during normal development will be an important prerequisite for developing new therapies for breast cancer.

## Paracrine signaling in breast cancer

Although the genetic and epigenetic landscape of a tumor is immensely different than that of its normal counterpart, the cellular heterogeneity exhibited in tumors is strongly influenced by cell-cell and paracrine interactions. The concept that these developmental cues are intact and active in the context of cancer is highlighted by studies involving established breast cancer cell lines and cells from patient tumors that display heterogeneity and respond to both autocrine and paracrine mediators (Figure 1B). For example, MCF-7 cells contain a small population of ER^-^ CD44^+^/CD24-ESA^+^ stem-like cells which can be expanded through an estrogen-induced paracrine FGF/FGFR/Tbx3 signaling pathway [42]. These authors concluded that ‘breast cancer stem cells (CSCs) are stimulated by estrogen through a signaling pathway that similarly controls normal mammary epithelial stem cell biology’. In studies using both established and primary ER^+^ primary patient-derived cell lines, both the epidermal growth factor and Notch signaling pathways have also been shown to operate downstream of estrogen in the regulation of ER^-^ CSCs [43]. Cytokine-induced and epigenetically regulated NF-κB (nuclear factor kappa-light-chain-enhancer of activated B cell) signaling in non-CSCs in triple-negative breast cancer cell lines was shown recently to induce jagged-1, which was then able to stimulate Notch signaling in CSCs [44]. Other studies using primarily immortalized and transformed human mammary epithelial cells, as well as primary MECs isolated from reduction mammoplasties, revealed both paracrine and autocrine effects of the Wnt and transforming growth factor-beta pathways on the interconversion between primary stem cell/progenitor cell-enriched (basal) and lineage-restricted (luminal) MECs [45]. These studies again support the hypothesis that both normal and neoplastic epithelial cells use very similar stem cell programs. They also illustrate the need to study the properties of individual cells and not total cell populations when performing both high-throughput genetic and small-molecule screens using breast cancer cell lines, which is now feasible with high-content microscopy.

## Conclusions

The above studies highlight the conservation of paracrine signaling pathways in mice and women as well as in normal mammary gland development and breast cancer. In normal development, precisely controlled positive and negative feedback pathways regulate the level and duration of signaling. However, in breast cancer, these feedback loops are often disrupted, leading to sustained and inappropriately regulated signaling. The ability to model and dissect these pathways genetically will be important for future progress in devising new and improved combinatorial therapeutic approaches for breast and other solid cancers.

## Abbreviations

CSC: cancer stem cell; ER: estrogen receptor; FGFR: fibroblast growth factor receptor; IL: interleukin; JAG1: jagged-1; MaSC: mammary stem cell; MEC: mammary epithelial cell; PR: progesterone receptor; RANKL: receptor activator of nuclear factor kappa-B ligand; Smo: Smoothened.

## Competing interests

The authors declare that they have no competing interests.
